# Artificial Larval Diet Mediates the Microbiome of Queensland Fruit Fly

**DOI:** 10.3389/fmicb.2020.576156

**Published:** 2020-09-16

**Authors:** Rajib Majumder, Brodie Sutcliffe, Saleh Mohammad Adnan, Bishwo Mainali, Bernard C. Dominiak, Phillip W. Taylor, Toni A. Chapman

**Affiliations:** ^1^Applied BioSciences, Macquarie University, North Ryde, NSW, Australia; ^2^Biosecurity and Food Safety, NSW Department of Primary Industries, Elizabeth Macarthur Agricultural Institute (EMAI), Menangle, NSW, Australia; ^3^Department of Entomology, Bangladesh Agricultural University, Mymensingh, Bangladesh; ^4^Biosecurity and Food Safety, NSW Department of Primary Industries, Orange, NSW, Australia

**Keywords:** Tephritidae, gut bacteria, Illumina sequencing, development, domestication, mating, stress tolerance, artificial diet

## Abstract

Larval diets used for artificial rearing can have a significant effect on insect biology. The Queensland fruit fly (aka “Qfly”), *Bactrocera tryoni* (Froggatt) (Diptera: Tephritidae), is one of the greatest challenges for fruit growers in Australia. The sterile insect technique (SIT) is being developed to manage outbreaks in regions that remain free of Qfly and to reduce populations in regions where this species is endemic. Factory scale rearing is essential for SIT; however, artificial larval diets are known to affect the microbiome of Qfly, which may then affect fly performance. In this study, high-throughput Illumina sequencing was used to assess the Qfly microbiome in colonies reared, for five generations from nature, on two common artificial diets (carrot and gel). At generation five (G5), the microbiome was assessed in larvae, pupae, adult males and adult females and standard fly quality control parameters were assessed together with additional performance measures of mating propensity and survival under nutritional stress. At the genus level, bacterial communities were significantly different between the colonies reared on the two larval diets. However, communities converged at Phyla to family taxonomic levels. Bacterial genera of *Morganella, Citrobacter, Providencia*, and *Burkholderia* were highly abundant in all developmental stages of Qfly reared on the gel diet, when compared to the carrot diet. Despite abundance of these genera, a greater percentage of egg hatching, heavier pupal weight and a higher percentage of fliers were found in the Qfly reared on the gel diet. Mating propensity and survival under nutritional stress was similar for adult Qfly that had been reared on the two larval diets. Overall, our findings demonstrate that the artificial larval diet strongly influences the microbiome and quality control measures of Qfly, with likely downstream effects on performance of flies released in SIT programs.

## Introduction

Insects brought into the laboratory from nature and reared over multiple generations are confronted by a new environment that is very different from nature, and are exposed to significant selection pressures that lead to laboratory adaptation (“domestication”) (Chambers, 1977; Hoffmann et al., 2001). In tephritid fruit flies, adaptation to artificial rearing conditions has been reported to have significant influence on genetic diversity and numerous life history traits, including development, stress tolerance and reproductive behavior. Mass reared fruit flies tend to mature at a younger age than wild type flies and may have reduced sexual competitiveness or compatibility with wild populations and reduced environmental tolerance (Gilchrist et al., 2012; Zygouridis et al., 2014; Schutze et al., 2015; Pérez et al., 2018). These changes, resulting from domestication, are anticipated to have important implications for the success of the sterile insect technique (SIT), an environmentally benign pest management technique in which millions of sterile insects are released to induce reproductive failure in females of pest populations (Knipling, 1955; Hendrichs et al., 1995; Vreysen et al., 2006).

Tephritid fruit flies are amongst the world’s most economically damaging insect pests (Norrbom et al., 1999). SIT has proven an effective means to manage some of the most economically damaging fruit flies including Mediterranean fruit fly (Medfly) *Ceratitis capitata* (Wiedemann) (Reyes et al., 2007), melon fly *Zeugodacus cucurbitae* (Coquillett) (Kakinohana, 1994; Yosiaki et al., 2003), Oriental fruit fly *Bactrocera dorsalis* (Hendel) (Orankanok et al., 2007), and Mexican fruit fly *Anastrepha ludens* (Loew) (Orozco-Dávila et al., 2015). In Australia, SIT has been implemented to eradicate outbreaks of Queensland fruit fly (Qfly) *Bactrocera tryoni* (Froggatt) in regions where this species has not yet established, and to suppress established populations (Dominiak et al., 2003; Fanson et al., 2014). With increasing restrictions on the use of insecticides for fruit fly control due to concerns about environmental and human health (Dominiak and Ekman, 2013), there is a growing need for the development of viable alternatives. There has been substantial interest and investment in development of SIT as a sustainable and environmentally benign solution.

Insects, including tephritid flies, commonly host a large diversity of microbes that can influence insect health (Jurkevitch, 2011). Microbial communities are often highly abundant in insect digestive systems (Dillon and Dillon, 2004), especially bacteria (Broderick et al., 2004; Robinson et al., 2010). In many cases, symbiotic bacteria have been found to provide nutrition that contributes to insect host fitness (Baumann, 2005; Akami et al., 2019). Microbes can provide amino acids (Nogge, 1981) and essential vitamins (Douglas, 1998), as well as nitrogen and carbon compounds (Benemann, 1973; Dillon and Dillon, 2004) to insect hosts. Some gut microbiota may have the ability to alternate between mutualism/commensalism and parasitism in response to changes in host diet (De Vries et al., 2004). Confirming the importance of the insect microbiome, elimination of resident bacteria can sharply reduce fly fitness (Ben-Yosef et al., 2008; Dinh et al., 2019).

Some microbiota may be acquired through insect diet, and diet is hence a major exogenous factor that can directly influence the composition of the insect gut microbial community and its metabolic capabilities (Chandler et al., 2011; Broderick and Lemaitre, 2012; Mason et al., 2014; Yun et al., 2014; Majumder et al., 2019). Additionally, variation in the diet nutritional composition (protein, carbohydrate and lipids) can influence both the gut microbiome biodiversity and community structure (Broderick et al., 2004; Ravenscraft et al., 2019; Woruba et al., 2019). Increasing our knowledge of these relationships may identify ways to enhance the quality of artificial diets, with the goal of improving performance in laboratory or mass-reared insects. To date, only a handful of studies have analyzed the microbiome of tephritid fruit flies reared on artificial diet (Behar et al., 2008b; Andongma et al., 2015; Morrow et al., 2015; Yong et al., 2017a; Deutscher et al., 2018; Malacrinò et al., 2018; Ventura et al., 2018; Woruba et al., 2019, Koskinioti et al., 2020). To our knowledge, however, there are no studies directly comparing the effect of different artificial larval diets on the gut bacterial community of tephritid fruit flies across the developmental stages of larvae, pupae and adult.

Larvae of the highly polyphagous Qfly develop in diverse host fruits (Hancock et al., 2000; May and Drew, 2003). In Qfly, the bacterial microbiome is largely transmitted vertically from the mother to the offspring when eggs are laid (Deutscher et al., 2018; Majumder et al., 2019). Descriptions are available characterizing the bacteria associated with wild and domesticated Qfly larvae (Deutscher et al., 2018; Majumder et al., 2019), pupae (Fitt and O’Brien, 1985) and adult flies (Thaochan et al., 2010; Morrow et al., 2015; Woruba et al., 2019). However, the effects of larval diet on changes in the Qfly microbiome through the early stages of domestication are not known. Different types of traditional solid diet, which include a biological bulking agent such as wheat meal, dehydrated carrot or lucerne chaff, have been used for rearing Qfly (Finney, 1956; Jessup, 1999; Dominiak et al., 2002, 2008). Usually, carrot-based diets have been used in moderate scale rearing and lucerne chaff-based diets used in factory-scale rearing (Jessup, 1999; Fanson et al., 2014). However factory-scale rearing and some laboratories now use a gel larval diet (Moadeli et al., 2017, 2018a,b,c, 2020). Mainali et al. (2019) found that Qfly reared on the gel diet produce better quality flies compared to solid diets containing carrot or lucerne chaff. Previous studies have assessed the bacterial populations of Qfly larvae and Qfly adults reared on a carrot diet and lucerne chaff diet (Morrow et al., 2015; Deutscher et al., 2018; Woruba et al., 2019). However, there are no studies investigating the microbial communities at all developmental stages of the Qfly reared on a gel diet compared to other larval diets, or direct comparisons through the early stages of domestication.

In the present study, high-throughput Illumina sequencing is used to investigate bacterial diversity and abundance in the microbiome of laboratory-reared Qfly at different life stages. Two Qfly colonies were established from the same wild material and were maintained through five generations of laboratory rearing separately on carrot and gel larval diets. After five generations, the microbiome was assessed, along with key quality control and performance parameters. We found that bacterial communities were significantly different between flies reared on the two larval diets, although communities converged at family taxonomic levels. Gel diets resulted in better egg hatch, heavier pupal weight and a higher percentage of fliers. Mating propensity and survival under nutritional stress was similar for the two diets. This study greatly improves our understanding of how artificial diets affect the microbiome of laboratory reared flies, and has significant implications for factory-scale rearing in SIT programs.

## Materials and Methods

### Colony Origins

Colony was established in 2017 from infested fruits (Majumder et al., 2020). Briefly, infested Pomegranate *Punica granatum*, Green Apple *Malus pumila* and Quince *Cydonia oblonga* were collected from different geographic locations in the Australian states of New South Wales (NSW) and Victoria (VIC) (Table 1). The infested fruits were collected from under trees, and most were over-ripe. After collection, all fruits were stored in plastic bins (60L, 447 mm× 236 mm× 663 mm, Award, Bunnings Warehouse, Greenacre, NSW, Australia) containing a 1 cm deep layer of fine vermiculite (Grade 1, Sage Horticultural, Hallam, VIC, Australia) in a controlled environment laboratory (25 ± 0.20°C, 65 ± 3% RH and 11 h: 1 h: 11 h: 1 h light: dusk: dark: dawn photoperiod). Larvae emerged from the fruit and pupated in the vermiculite. Pupae were sieved from the vermiculite, combined, and placed in a cage to emerge (Megaview BugDorm 44545, 47.5 cm× 47.5 cm× 47.5 cm, MegaView Science Co., Ltd., Taichung, Taiwan). Approximately 600 adult Qfly were obtained and from this pool 300 flies were transferred to each of two mesh cages to establish the studied colonies (Megaview BugDorm 44545, 47.5 cm× 47.5 cm× 47.5 cm, MegaView Science Co., Ltd., Taichung, Taiwan). Adult flies were provided hydrolyzed yeast (MP Biomedicals, Cat. No. 02103304) and commercial sucrose (CSR^®^ White Sugar, Maribyrnong, VIC, Australia) separately, and water through a moist sponge.

**TABLE 1:** Fruit types and origin for wild *Bactrocera tryoni* larvae collection.

	**Fruit source and number**	
**Geographic location of collection**	**of fruits collected**	**Collection date**
Coomealla, NSW GPS: Lat. 34° 5′50.97″, Long. 142° 3′7.21″	Pomegranate 37 pieces	5/05/17
St. Germain’s, Between Tatura and Echuca in Victoria GPS: Lat. 36°10′48.86″, Long. 145° 8′50.74″	Green Apple 41 pieces	05/05/17
Downer road between Tatura and Toolamba in Victoria GPS: Lat. 26°38′34.92″, Long. 152°56′22.99″	Quince 52 pieces	05/05/17

*A total of six replicate larvae, and fruit flesh samples were collected from each fruit origin.*

### Colony Maintenance

We reared the two Qfly colonies on different artificial larval diets; carrot and gel (Moadeli et al., 2017; Mainali et al., 2019) (see Supplementary Tables S1, S2 for details, Supplementary Figure S1). The carrot diet was prepared by mixing all ingredients using a food mixer (KitchenAid, Model No: 5KSM150PSAER, United States) for 15 min (5 min slow and 10 min fast cycles) and kept at room temperature for 12–24 h before use. The gel diet was prepared as in Moadeli et al. (2017), by mixing all the dry ingredients using a blender (Kenwood, Multipro FPM810 series, China) for 5 min. Water was mixed with agar and the solution boiled. The boiled agar and the dry mixture were then mixed together. We transferred 150 g of carrot diet and 150 mL of gel diet into larvae rearing containers (17.5 cm long, 12 cm wide and 4 cm deep) (Castaway food packaging, Arndell Park, NSW, Australia).

At each generation, eggs were collected using an oviposition device comprising a 300 mL semi-transparent white soft plastic bottle (low density polyethylene). The oviposition device had numerous ∼1 mm holes through which females could oviposit, and contained 20 mL of water to maintain humidity and a few drops of natural apple juice to attract the female flies and encourage egg laying (Collins et al., 2008). Eggs were collected from 14–16 day old mature flies between 9 am and 3 pm on a single day. The oviposition device was rinsed with distilled water to wash out the eggs. The eggs were then collected using a 50 mL falcon tube and 250 μL of eggs in suspension which was transferred to the larval diet using a 1000 μL pipette (ca. 3500 eggs, ca. 23 eggs per gram of diet) (Moadeli et al., 2017). The larval rearing containers were then covered with plastic lids until the larvae reached their third instar and exited the diet to pupate. The rearing trays were then placed in a container with a 1 cm layer of fine vermiculite on the bottom. The larvae exited the rearing container and landed in the vermiculite where they pupated. Pupae were collected after sifting them from the vermiculite. In order to establish the two colonies, approximately 2000 pupae from each diet were placed in a mesh cage (Megaview BugDorm 44545, 47.5 cm× 47.5 cm× 47.5 cm, MegaView Science Co., Ltd., Taichung, Taiwan) for emergence and the rearing protocol was followed for producing subsequent generations until G5. From the colony (G5) of each type of diet, third instar larvae (*N* = 12), 8 days old pupae (*N* = 12) and 15 days old sexually mature male (*N* = 12) and female adult flies (*N* = 12) were collected for sequencing.

### Sample Preparation

For sample processing, Qfly larvae, pupae and adult flies (male and female separately) from the G5 colonies were surface sterilized using 0.5% Tween 80 (Sigma-Aldrich, St. Louis, MO, United States, Cat. No. 9005656), 0.5% Bleach (Sodium hypochlorite) (Sigma-Aldrich, St. Louis, MO, United States, Cat. No. 7681529) and 80% Ethanol (Sigma-Aldrich, St. Louis, MO, United States, Cat. No. 65175) for 30 s, and rinsed 3 times in 1M sterile phosphate-buffered saline (1x PBS) again for 30 s. The PBS from the 2nd and 3rd washes were kept and 100 μL spread-plated on to five types of microbial growth medium (de Man, Rogosa and Sharpe Agar, Tryptone Soya Agar, MacConkey Agar, Potato Dextrose Agar and Yeast-dextrose Agar medium) (Sigma-Aldrich, St. Louis, MO, United States) to check the performance of the sterilization method. All plates were incubated at 32 and 35°C for 24 to 48 hr (Majumder et al., 2019, 2020). Immediately after the sterilization process, the guts of adult flies were dissected using a stereomicroscope (Leica MZ6 stereo-microscope, Leica^®^, Wetzlar, Germany). Using sterile pestles, larvae, pupae, and dissected guts from the adults were homogenized separately in a solution of Brain Heart Infusion (BHI) broth (Oxoid Ltd., Basingstoke, United Kingdom, Lot # 1656503) and 20% Glycerol (Sigma Aldrich, St. Louis, MO, United States, Lot # SHBG2711V) and each sample was stored in a separate cryovial tube (Simport Scientific, Saint-Mathieu-de-Beloeil, QC, Canada). All the samples are preserved at −80°C. All procedures were completed in a sterile environment (Biological air clean bench, safe 2020 1.2, Thermo Scientific, Dreieich, Germany).

### Microbiome Profiling

DNeasy Power Lyzer Power Soil Kit-100 (Qiagen, Hilden, Germany, Cat. no. 12855-100) was used for the DNA extraction following the manufacturer’s protocol. DNA extracts were then quantified in the Invitrogen^TM^ Qubit^®^ dsDNA High Sensitivity (HS) Assay Kit (Life Technologies, Eugene, OR, United States). PCR amplification and sequencing were performed by the Australian Genome Research Facility, University of Adelaide, Plant Genomics Centre, Hartley Grove, URRBRAE, SA 5064, AU. For bacterial identification, the V1-V3 16S rRNA region was amplified using primers 27F (5′AGAGTTTGATCMTGGCTCAG-3′) and 519R (3′ GWATTACCGCGGCKGCTG-5′) (Lane, 1991) as used previously in Majumder et al. (2019, 2020). Reactions contained 1X AmpliTaq Gold 360 mastermix (Life Technologies, Eugene, OR, United States), 0.20 μM of each forward and reverse primer and 25 μL DNA. PCR cycling conditions consisted of denaturation at 95°C for 7 min, 35 cycles of 94°C for 45 s, 50°C for 60 s and 72°C for 60 s, and a final extension of 72°C for 7 min. A second PCR was used to adhere sequencing adaptors and indexes to the amplicons. Primestar max DNA Polymerase was used to generate a second PCR amplicon (Takara Bio Inc., Shiga, Japan; Cat. No. #R045Q). The resulting amplicons were measured using a fluorimeter (Thermo Fisher Scientific, North Ryde, NSW, Australia) and normalized (Fouts et al., 2012). The normalized samples were pooled and quantified by qPCR prior to sequencing (Kapa qPCR Library Quantification kit, Roche, Basel, Switzerland). The resulting amplicon library was then sequenced on the Illumina MiSeq platform (San Diego, CA, United States) with 2 × 300 base pairs paired-end chemistry (Caporaso et al., 2010).

### Sequence Data Processing

The Greenfield Hybrid Amplicon Pipeline (GHAP) was used to process bacterial 16s rRNA amplicon sequences (Greenfield, 2017; Sutcliffe et al., 2018). The GHAP is a publically available amplicon clustering and classification pipeline^1^ (Greenfield, 2017) built around tools from USEARCH (Edgar, 2010) and the Ribosomal Database Project (RDP) (Maidak et al., 1996), combined with locally written tools for demultiplexing, trimming and generating OTU (Operational Taxonomic Unit) table. This hybrid pipeline produces a table of taxonomically assigned OTUs and their associated reads counts across all samples. First, the amplicon reads were demultiplexed and trimmed, and the read pairs were then merged (using *fastq_mergepairs*) and de-replicated (using *fastx_uniques*). The merged reads were then trimmed again and clustered at 97% similarity (using *cluster_otus*) to generate OTUs. Representative sequences from each OTU were then classified both by finding their closest match in a set of reference 16S sequences (using *usearch_global*), and by using the RDP Naïve Bayesian Classifier. The pipeline mapped the merged reads back onto the classified OTU sequences to get accurate read counts for each OTU/sample pairing and generated an OTU table complete with taxonomic classifications and species assignments. The OTU table was then summarized over all taxonomic levels, combining the counts for identified taxa across all OTUs. The pipeline finally classified all the merged reads using the RDP Classifier, regardless of whether they were assigned to an OTU. This last step was done to provide confidence in the clustering and OTU formation steps by providing an independent view of the community structure.

All OTUs that were assigned to “Mitochondria” at the Order level were removed from the dataset before downstream processing. The above biome table was rarefied to 10,000 reads per sample, repeating this 50 times and averaging the counts to obtain a representative rarefaction to maintain equal sequence depth among all samples. This was achieved using an in-house python script. Those samples with <10,000 reads were excluded. The data were then normalized as the percentage of relative abundance, and are henceforth referred to as the OTU table (Supplementary File S1). All the figures of bacterial relative abundance at different developmental stages and between generations in colonies reared on different diets were plotted in Prism 8 [version 8.0.1 (145), GraphPad software, Inc] as used previously in Majumder et al. (2019, 2020). The Illumina sequence data were deposited in NCBI database under Bioproject PRJNA647614.

### Quality Control Measures

Quality control assessment was based on standard procedures (Collins et al., 2008; FAO/IAEA/USDA, 2014; Moadeli et al., 2017; Adnan et al., 2018; Mainali et al., 2019).

### Egg Hatching

A 100 μL pipette was used to collect the eggs which were counted under a stereomicroscope (Leica MZ6, Wetzlar, Germany) on moistened black filter paper using a soft paintbrush to move the eggs. Five sets of 50 eggs from G5 of each Qfly colony were transferred onto a 1 cm × 3.5 cm strip of moistened black filter paper. Twenty-five milliliters of gel diet and 25 g of carrot diet were poured separately into 90 mm Petri dishes (see Supplementary Figure S1) with five replicates for each diet. The filter paper with the eggs was laid on top of the larval diet. Petri dishes were covered and kept in the controlled environment room. Four days later, the number of the unhatched eggs was counted. The percentage of egg hatch was calculated as {[N of egg hatched/(N unhatched + N hatched)] × 100}. The data from egg hatching rate were used to calculate pupal recovery rate.

### Pupal Recovery

Twenty-five milliliters of gel diet and 25 g of carrot diet were poured separately into 90 mm Petri dishes with five replicates for each diet. Eggs were collected and handled as described above. We counted 50 eggs and immediately transferred them to the diet surface. All Petri dishes were placed in the laboratory environment with lids on. The lids were removed when larvae were ready to pupate. Plates were then placed into separate plastic containers (12 L) containing a 1 cm deep layer of fine vermiculite (Grade 1, Sage Horticultural, Hallam, VIC, Australia) and with a mesh lid. The pupae were collected every 2 days. Pupal recovery was calculated as the total number of pupae divided by the number of hatched eggs multiplied by 100.

### Pupal and Adult Weight

A microbalance (Sartorius ME5, Germany) was used to assess weight of pupae and adults. Thirty pupae were collected from each colony and individually weighed on the ninth day from the day of first pupation and mean weight was calculated. To assess adult fly weight, approximately 300 pupae from each colony were placed in a mesh cage (Megaview BugDorm 44545, 47.5 cm× 47.5 cm× 47.5 cm, MegaView Science Co., Ltd., Taichung, Taiwan) for emergence. No food or water were supplied in the cages. Few flies emerged on the first day and these were discarded. On the second day of emergence, the pupae were transferred to new cages and flies that emerged over the following 2 h were collected and sexed. Thirty adult male and 30 adult females were collected from each colony, placed in a −20°C freezer and individually weighed to calculate the mean weight.

### Flight Ability

Adult fly emergence, percentage of fliers, the rate of fliers and the proportion of male flies was assessed using standard flight ability assays (Moadeli et al., 2017). Two days before fly emergence, 100 pupae (8 days old, five replicates from each diet) were placed separately in 55 mm Petri dishes without lids. The dishes containing the pupae were placed in the center of 90 mm Petri dish lids with black filter paper on the base. A black 100 mm tall acrylic flight ability tube (89 mm external diameter, 84 mm inner diameter) was placed over the 90 mm Petri dish lid. Fine talcum powder on the tube interior prevented the emerged flies from walking out. The whole setup with pupae was placed in a mesh cage (dimensions 32.5 cm× 32.5 cm× 32.5 cm size, Megaview BugDorm- 43030F, MegaView Science Co., Ltd., Taichung, Taiwan) in the laboratory beneath 20-watt fluorescent lights (ca. 1250 lx at the top and ca. 900 lx at the base of the flight ability tubes). A flyback tube was placed 6 cm away from the flight ability tube. During fly emergence, we removed the flies that flew out from the tube every day in the morning and afternoon to minimize flyback. All collected flies were stored in a −20°C freezer for later assessment. We collected and counted the flies until all emergence was complete (4–5 days) and categorized them as; (1) not emerged (inside unopened pupal case); (2) partially emerged (a portion of adult body remaining stuck in the puparium); (3) deformed (fully shed the pupal case but with deformed or damaged wings or appendages); (4) non-fliers (morphologically normal flies in the flight tube minus the number of flies in the flyback tube); (5) fly-back (the number of flies inside the fly-back tube plus the same number of normal flies inside the flight tube); and (6) fliers (the number of flies that were collected from outside the tubes plus fly-back). Assessed metrics included:

• *Percentage of adult emergence:* [N pupae – (N not emerged + N partially emerged)/N Pupae] × 100.
• *Percentage of fliers:* [N pupae – (N not emerged + N partially emerged + N deformed + N non-fliers)/N pupae] × 100.
• *Rate of fliers:* percentage of fliers/percentage of emergence × 100.

### Mating Performance

For mating trials, approximately 400 pupae from each colony were placed in a mesh cage (Megaview BugDorm 44545, 47.5 cm× 47.5 cm× 47.5 cm, MegaView Science Co., Ltd., Taichung, Taiwan) for emergence. After emergence, cages were supplied with water-soaked cotton wool in a 70 mL sample container. Food was provided separately as dry granular sucrose (CSR^®^ White Sugar, Maribyrnong, VIC, Australia) and yeast hydrolyzate (MP Biomedicals, Irvine, CA, United States, Cat. No. 02103304) (3:1) on two 90 mm Petri dishes *ad libitum*. To obtain mature flies (12–16 day old) to pair with experimental flies, ca. 400 pupae from a separate laboratory colony (reared on gel larval diet for >25 generations) were placed in separate mesh cages (Megaview BugDorm 44545, 47.5 cm× 47.5 cm× 47.5 cm, MegaView Science Co., Ltd., Taichung, Taiwan) for adult emergence. Similar to flies from the experimental colonies, these flies were supplied with water-soaked cotton wool in a 70 mL sample container, dry granular sucrose and yeast hydrolyzate as food on a 90 mm Petri dish *ad libitum*. All cages of flies were sorted according to sex within 3 days after emerging by collecting and transferring individual flies in glass tubes to clear plastic 12 L cages that had a mesh-covered ca. 80 cm^2^ window for ventilation. Approximately 160 flies were sorted into each 12 L cage. No calling, courting, or mating was observed in cages prior to separating the sexes.

Mating trials were conducted when flies were sexually mature (12–16 day old). Qfly mate at dusk (Tychsen and Fletcher, 1971). Four hours before the onset of dusk, 40 males and 40 females from each experimental group were placed individually in clear plastic 1.25 L containers with a mesh-covered window (ca. 28 cm^2^) for ventilation. Each fly was individually paired with a sexually mature (12–16 day old) fly of the opposite sex. Virgin flies of this age fed a diet of sugar and yeast hydrolyzate show a high level of sexual receptivity (Vijaysegaran et al., 2002; Pérez-Staples et al., 2007). Periodic observations were carried out after pairs were set up, and continuous observations began 90 min prior to the onset of dusk. The time of onset of copulation for each mating pair was recorded to assess mating latency (time from the start of dusk until the onset of mating, in minutes) and observations continued until the last pair had separated to assess mating duration for each mating pair.

### Survival Under Nutritional Stress

Pupae were placed in separate mesh cages (Megaview BugDorm 44545, 47.5 cm× 47.5 cm× 47.5 cm, MegaView Science Co., Ltd., Taichung, Taiwan) for adult emergence. Few flies usually emerge on the first day of emergence, and these were discarded. On the second day of emergence, pupal trays were transferred to new cages and flies that emerged in the following 2 h were used for the study. Forty females and 40 males were placed in individual, 5 mL round bottom plastic vials (Lab Australia Pty Ltd., NSW, Australia). Flies were given no access to food or water after being placed in the vials until death. The number of dead flies was recorded by visually inspecting the vials every 3 h. Flies were considered dead when they were incapable of holding onto the inner surface of the plastic vial, and when no movement of their legs or mouthparts was observed after the vials were gently flicked with a finger. Dead flies were removed at each assessment.

### Statistical Analysis

#### Microbiome Analysis

The OTU table was imported into Primer-E v7 for analysis as described in Clarke and Ainsworth (1993); Sutcliffe et al. (2017), Majumder et al. (2019, 2020). In brief, all statistical testing was performed on fixed factors associated with developmental stage and sex (larvae, pupae, adult male and female) from which 12 replicates were collected. The DIVERSE function was used to generate univariate biodiversity metrics, species richness, Pielou’s evenness and Shannon’s and Simpson’s biodiversity indices. Statistical differences between these metrics were assessed in JMP Statistical Software Version 10.0.0 (SAS Institute, Cary, NC, United States) using one-way analysis of variance (ANOVA) and Tukey-Kramer *post hoc* analysis. The Operational Taxonomic Unit (OTU) table was first log transformed using Primer-E v7 to observe the taxonomic compositional changes for the bacterial communities. A Bray-Curtis similarity matrix was derived from this transformed data and a permutation analysis of variance (PERMANOVA) pairwise comparison was conducted to compare all community samples. A *p*-value of <0.05 was considered statistically significant. Further, ordination plots of these communities were visualized using principal coordinates analysis (PcoA) in Primer-E.

We performed ANOVA and *post hoc* Tukey-Kramer tests to determine whether significant differences occurred in the relative abundance of bacterial communities at all developmental stages of the Qfly, from carrot and gel diet reared colonies. We used Benjamini–Hochberg to correct for multiple testing false-discovery rate (FDR) and an alpha threshold of 0.05 on FDR corrected ANOVA *p*-values was used to determine significance.

#### Quality Control, Mating and Survival Under Nutritional Stress

All analyses were performed using JMP statistical software (Version 10.0.0, SAS Institute, Cary, NC, United States). Qfly quality control measures were analyzed with ANOVA and pair-wise Student’s *t*-tests. Prior to the analysis, distribution patterns were observed for all quality control data. Figures of quality control measures were plotted using Prism 8 software (1995–2018 GraphPad software, Inc., United States).

Mating probability (binary outcome) was assessed using nominal logistic regression with significance tested using likelihood ratio tests (*G*-test). Main effects included in the model were diet (nominal) and sex (nominal). Model parameter estimates were inspected to identify effects. Mating latency, mating duration and survival under nutritional stress (continuous outcomes) were analyzed for each treatment using least squares regression (2-way ANOVA) including diet (nominal) and sex (nominal). Initially, all interactions were included, and a backward model selection process was performed by removing non-significant interactions.

## Results

### 16s rRNA Sequence Reads and OTUs

We sequenced the bacterial microbiome of 96 Qfly samples from G5 reared on the carrot and gel diet. This included larvae (*N* = 12), pupae (*N* = 12), adult males (*N* = 12) and adult females (*N* = 12) from each diet. Among them, 79 were retained after quality control and rarefaction at 10,000 reads per sample (9 and 8 samples were removed from carrot and gel diet, respectively). After rarefaction and quality control, a total of 287 bacterial OTUs were detected across the 79 samples (Supplementary File S1).

### Gut Bacterial Diversity

**FIGURE 1:**
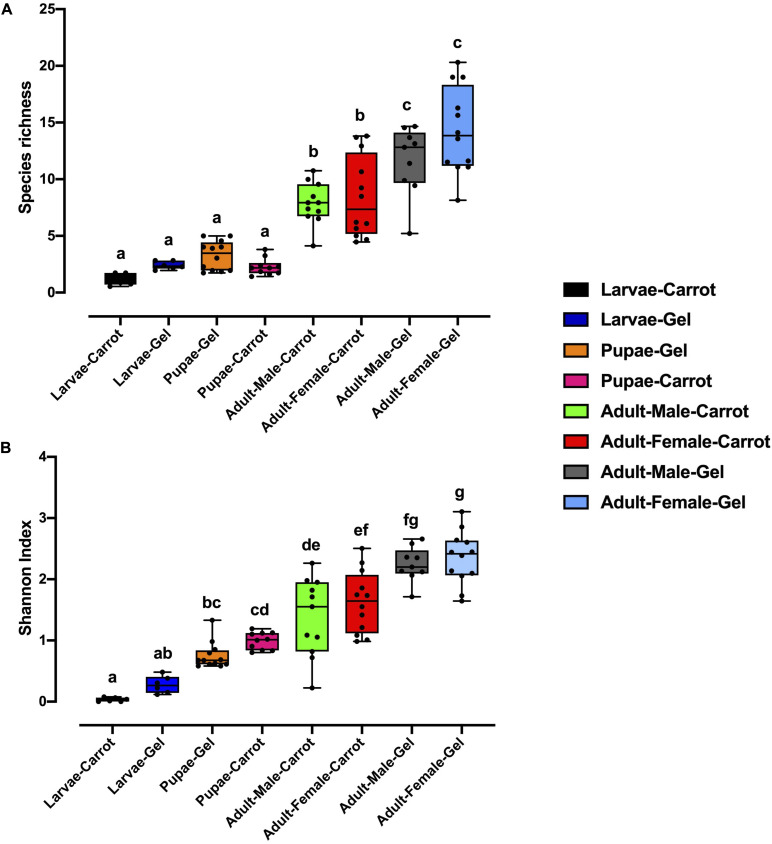
Alpha diversity of the bacterial microbiome of the *B. tryoni* developmental stages at G5 reared on two different artificial diets, **(A)** Species richness **(B)** Shannon index. Different letters indicate significant Tukey’s *post hoc* comparisons (*P* < 0.05).

**FIGURE 2:**
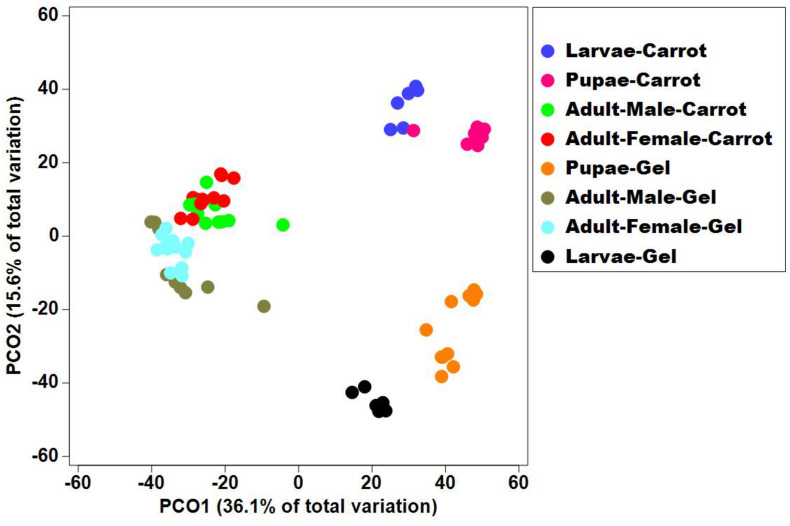
Principal co-ordinate analysis of the bacterial communities in the Qfly developmental stages at G5 reared on carrot diet and gel diet.

Bacterial alpha diversity metrics, species richness and Shannon biodiversity indices, were calculated for each Qfly developmental stage from the two larval diets (Figures 1A,B). Biodiversity metrics were insensitive to larval diet, with no significant differences at any developmental stage (Figures 1A,B). In contrast, beta diversity showed significant differences between diets at all developmental stages (PERMANOVA; *p* < 0.05) (Supplementary File S1). Principal coordinates analysis (PCoA) of Bray-Curtis similarity matrix was plotted to visualize variation in bacterial communities (Figure 2). Based on the PCoA ordination plot, we inferred that each developmental stage of the Qfly, both from carrot and gel diet, had a distinct microbiota population. In the PCoA scatter plot, PCO1 captured 36.1% of the total variance in the dataset and corresponded with the separation of larval and pupae samples from those of the adult (Figure 2). The second axis, PCO2 captured a further 15.6% of variance in the data, and corresponded with the separation of communities associated with Qfly reared on different diets (Figure 2).

### Bacterial Communities of Qfly From Carrot and Gel Based Artificial Diets

The bacterial taxa detected in the larval, pupal and adult (both male and female) stages of Qfly reared on the carrot and gel diet represented a total of 5 phyla, 12 classes, 56 families, and 115 genera (Supplementary File S1). At the phylum level, we detected very little difference between the two diets, or across developmental stages. For example, Proteobacteria represented ∼ 90% of all microbial communities. One exception to this was the decrease of Actinobacteria in larvae, with an average relative abundance of 3.8% for those reared on the carrot diet, compared to 0.05% on the gel diet.

**FIGURE 3:**
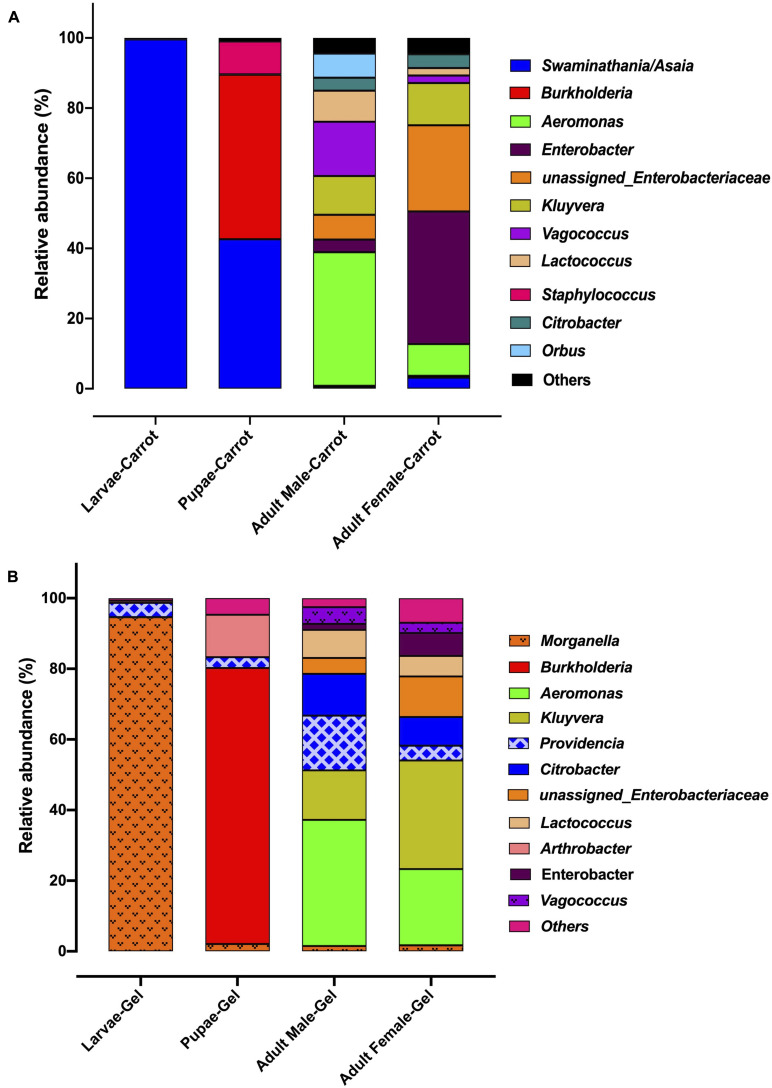
The relative abundance of the bacterial microbiota in Qfly at different developmental stages from generation 5 reared on **(A)** Carrot based larval diet, **(B)** Gel based larval diet.

**FIGURE 4:**
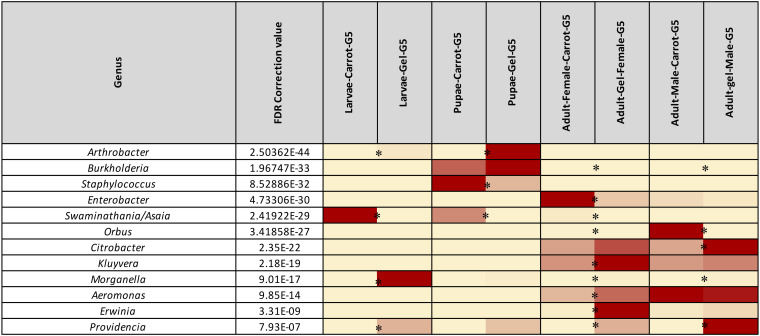
Heatmap representing the average relative abundance of the 12 most abundant (>1%) bacterial genera of Qfly from the carrot and gel-based diet groups on 16S rRNA gene amplicon data. Relative abundances for each genus are represented by a color on a spectrum from yellow to deep red. Lowest values are highlighted yellow, highest are represented as a deep red. Asterisks (*) indicate relative abundances of each developmental stage is significantly different between the carrot and gel diet.

We observed strong taxonomic trends at the family and genus level when comparing the two diets across developmental stages. In the larval stage, the relative abundance of Enterobacteriaceae was ∼ 99% for those reared on the gel diet. In contrast, the relative abundance of this family was <0.1% for those reared on the carrot diet (Supplementary File S1). Instead, the microbiomes of the carrot diet fed larvae were dominated by Acetobacteraceae, which represented almost 100% of the population, but was almost undetectable in the gel fed larvae (0.01%). These trends were mirrored at the genus level, with *Morganella* and *Providencia* (Enterobacteriaceae) being significantly more abundant in the larvae reared on the gel diet compared with those feed the carrot diet (Figures 3, 4 and Table 2). *Swaminathania/Asaia* (Acetobacteraceae) accounted for ∼ 99.9% of the larval microbiome for colonies fed on the carrot diet, but was only a minor component (<1%) of microbiomes from gel fed larvae (Figure 4).

**TABLE 2:** Taxonomic identification of the of the 14 most abundant bacterial genus in the Qfly from carrot and gel diet.

**Phylum**	**Class**	**Order**	**Family**	**Genus**	**% Carrot diet**	**% Gel diet**
Actinobacteria	Actinobacteria	Actinomycetales	Micrococcaceae	*Arthrobacter*	0.00	3.14
Proteobacteria	Betaproteobacteria	Burkholderiales	Burkholderiaceae	*Burkholderia*	12.02	19.55
Firmicutes	Bacilli	Bacillales	Staphylococcaceae	*Staphylococcus*	2.35	0.57
Proteobacteria	Gammaproteobacteria	Enterobacteriales	Enterobacteriaceae	*Enterobacter*	10.36	2.06
Proteobacteria	Alphaproteobacteria	Rhodospirillales	Acetobacteraceae	*Swaminathania/Asaia*	36.40	0.00
Proteobacteria	Gammaproteobacteria	Orbales	Orbaceae	*Orbus*	1.73	0.00
Proteobacteria	Gammaproteobacteria	Enterobacteriales	Enterobacteriaceae	unassigned_Enterobacteriaceae	7.93	4.00
Proteobacteria	Gammaproteobacteria	Enterobacteriales	Enterobacteriaceae	*Citrobacter*	1.91	4.99
Firmicutes	Bacilli	Lactobacillales	Enterococcaceae	*Vagococcus*	4.40	1.89
Proteobacteria	Gammaproteobacteria	Enterobacteriales	Enterobacteriaceae	*Kluyvera*	5.77	11.19
Proteobacteria	Gammaproteobacteria	Enterobacteriales	Enterobacteriaceae	*Morganella*	0.00	24.98
Firmicutes	Bacilli	Lactobacillales	Streptococcaceae	*Lactococcus*	2.75	3.44
Proteobacteria	Gammaproteobacteria	Aeromonadales	Aeromonadaceae	*Aeromonas*	11.79	14.32
Proteobacteria	Gammaproteobacteria	Enterobacteriales	Enterobacteriaceae	*Providencia*	0.00	6.70

*Percentage of the carrot and gel diet represents the percentage of the average relative abundance of each genus present in the Qfly sample from the two different diets.*

*Morganella, Providencia*, and *Swaminathania/Asaia* were also detected in the pupae and adults, and showed similar trends to those in the larvae. Specifically, *Swaminathania/Asaia* had a significantly higher relative abundance in pupae and adults reared on the carrot diet (42%) compared with those reared on gel diet (<0.01%; FDR corrected *p* < 0.0001). *Morganella* and *Providencia* were more abundant in the colony reared on the gel diet. These differences in the bacterial genera from Enterobacteriaceae were statistically significant for larvae and adults (FDR corrected *p* < 0.0001), but not for pupae.

Another notable trend within the pupal stage (Figure 3) was the higher relative abundance of the Micrococcaceae in the pupae from the gel diet colony (14%), compared with the carrot diet colony (<1%). As with the Acetobacteraceae, this trend was driven by a single genus, *Arthrobacter* (Figure 4). This genus was more abundant in the pupal developmental stage compared with the larvae, but it was also significantly more abundant in the larvae fed on a gel diet (0.5% vs. 0%, FDR corrected *p* < 0.0001, Figure 4). In addition, although *Burkholderia* was highly abundant in the pupal stage, significant greater abundance was detected both in adult males and females reared on the carrot diet compared to the gel diet (FDR corrected *p* < 0.0001, Figure 4).

In the adult stage, a number of diet-associated differences were detected that were unique to this developmental stage and/or the sex of adults (Figure 3). For example, in both male and female adults, *Orbus* was significantly more abundant in colonies reared on the carrot diet (FDR corrected *p* < 0.0001, Figure 4). However, it is notable that this genus was much more abundant in males than in females. Several additional diet associated differences were only evident in one adult sex. For example, *Kluyvera, Aeromonas*, and *Erwinia* were all significantly more abundant in adult females from the gel diet colony, compared with those from the carrot diet colony, but no differences were found in males. Conversely, *Citrobacter* was significantly more abundant in adult males of the gel diet colony than the carrot diet colony, but no differences were evident in females (Figure 4).

### Quality Control Measures

#### Percentage of Egg Hatch and Pupal Recovery

The percentage of egg hatching was not significantly different (*F*_1_,_8_ = 2.82, *p* = 0.132) between the carrot diet (66.80% ± 1.86 SE) and gel diet (71.20% ± 1.86 SE). No significant difference (*F*_1_,_8_ = 1.73, *p* = 0.225) was observed in the pupal recovery rate of the colony reared on carrot diet (58% ± 3.02 SE) and the colony reared on gel diet (63.60% ± 3.02 SE).

#### Weight

Pupae were significantly heavier (*F*_1_,_58_ = 62.16, *p* < 0.001) in the colony reared on from the gel diet (14.39 mg ± 0.23 SE) than in the colony reared on carrot diet (11.72 mg ± 0.39 SE). No significant difference (*F*_1_,_58_ = 0.07, *p* = 0.792) was found in the adult body weight of males reared on the carrot (7.91 mg ± 0.16 SE) and the gel diet (7.85 mg ± 0.16 SE). In contrast, adult body weight was significantly greater (*F*_1_,_58_ = 13.89, *p* < 0.001) in females reared on carrot diet (9.09 mg ± 0.14 SE) compared to the gel diet (8.36 mg ± 0.14 SE).

#### Flight Ability

There was no significant difference (*F*_1_,_8_ = 2.86, *p* = 0.129) in the percentage of adult emergence in Qfly reared on carrot diet (82 ± 1.67 SE) and gel diet (86 ± 1.67 SE). In contrast, the percentage of fliers was significantly higher (*F*_1_,_8_ = 5.389, *p* = 0.0488) for Qfly reared on gel diet (80.80 ± 1.95 SE) than on carrot diet (74.40 ± 1.95 SE).

#### Mating Propensity

Mating propensity did not vary significantly between carrot and gel diet reared flies (G_1_ = 0.02, *p* = 0.87). Also, there was no significant difference between the two diets in mating latency (*F*_1_,_51_ = 0.66, *p* = 0.42) or mating duration (*F*_1_,_51_ = 2.2, *p* = 0.14). Sex of the flies did not influence mating propensity (G_1_ = 0.02, *p* = 0.87), mating latency (*F*_1_,_51_ = 2.97, *p* = 0.09) or mating duration (*F*_1_,_51_ = 1.63, *p* = 0.20).

#### Survival Under Nutritional Stress

Survival of the flies that were subjected to nutritional stress did not differ significantly between flies reared on the carrot and the gel diet (*F*_1_,_156_ = 0.05, *p* = 0.82). In addition, survival under stress did not vary with the sex of flies (*F*_1_,_156_ = 0.25, *p* = 0.62).

## Discussion

The present study provides a comprehensive analysis of how two common artificial diets affect the microbiome of Qfly through the early stages of domestication across all developmental stages, along with key quality control and performance parameters. In a previous study, Majumder et al. (2020) used high-throughput Illumina sequencing to identify and characterize the microbial communities present in the different developmental stages of wild type Qfly (G0) at the point of entry into laboratory rearing. In the present study, we are able to address meaningful questions regarding how microbial communities change after five generations of rearing on artificial larval diets. Artificial larval diet strongly modulated the microbial community structure (beta diversity) across all developmental stages, but did not affect total biodiversity (as assessed by alpha diversity metrics: species richness and Shannon’s diversity index). Previously, the bacterial phyla Proteobacteria and Firmicutes have commonly been reported in Qfly larvae and adults in domesticated colonies reared on a carrot diet (Deutscher et al., 2018; Majumder et al., 2019; Woruba et al., 2019), and in other fruit flies including *Bactrocera neohumeralis*, *Bactrocera jarvisi*, *Bactrocera cacuminata, Bactrocera oleae, Zeugodacus tau, A. ludens, Anastrepha obliqua, Anastrepha serpentina*, *Anastrepha striata*, and *C. capitata* (Morrow et al., 2015; Malacrinò et al., 2018; Ventura et al., 2018; Koskinioti et al., 2020; Noman et al., 2020). Our results are consistent with these previous findings. However, it is noteworthy that variations in diet and developmental stage had a limited impact on microbial community composition at the phylum level. Analyses at the family and/or genus level are far more informative of such variations in Qfly populations.

The fruit fly microbiome changes markedly across developmental stages (Andongma et al., 2015; Yong et al., 2017a,b; Noman et al., 2020). This is consistent with the magnitude of metabolic and physiological change that occurs during metamorphosis. Despite the substantial changes in the overall microbiome across developmental stages, some bacterial families and genera showed consistent trends at all developmental stages when comparing artificial diets. Specifically, the gel diet was associated with an increased relative abundance of the Enterobacteriaceae genera *Morganella* and *Providencia*, while these taxa were almost non-existent in colonies reared on the carrot diet (Figure 3). Conversely, the carrot diet resulted in an increased abundance in the Acetobacteraceae genus *Swaminathania/Asaia*, which was found only at very low abundance in the colonies reared on the gel diet. Previous studies of wild Qfly larvae (Deutscher et al., 2018; Majumder et al., 2019, 2020) and adults (Woruba et al., 2019; Majumder et al., 2020) suggested that Enterobacteriaceae and Acetobacteraceae are naturally abundant in the Qfly microbiome. In contrast, the response of some taxa to diet was limited to a specific developmental stage. This included the bacterial genera *Orbus* and *Enterobacter*, which were highly abundant in adult males and females, respectively, from the carrot diet colony. Conversely, *Kluyvera, Aeromonas*, and *Erwinia* were highly abundant in adult males from the gel diet colony. Similarly, Woruba et al. (2019) found that field-collected male and female Qfly had significant differences in bacterial diversity and in bacterial composition. In contrast, we observed that the bacterial community of larvae reared on a carrot and gel diet were clustered at a substantial distance in PCoA ordination plot (Figure 2). A possible explanation is that the main ingredient of the diet was plant based in both nature and the carrot diet reared laboratory colonies.

Diet is a key factor influencing the gut microbiome of fruit flies including *Bactrocera, Ceratitis, and Anastrepha* (Malacrinò et al., 2018; Ventura et al., 2018; Andongma et al., 2019; Majumder et al., 2019). Diet effects on the microbiome are also commonplace in other insects, for example: cotton bollworm (*Helicoverpa armigera*) (Xiang et al., 2006), the ground dwelling beetle (*Coleoptera*) (Kudo et al., 2019), gypsy moth (*Lymantria dispar*) (Broderick et al., 2004) and *Drosophila* (Colman et al., 2012). In our study, the starting material, rearing environments, adult diet and the generations were the same for the colonies reared on the two tested larval diets. This single difference of larval diet resulted in substantial variation in the bacterial composition across all developmental stages. In both the carrot diet and the gel diet, the antimicrobial agents of sodium benzoate and citric acid were common (Mainali et al., 2019), but the antifungal Nipagin (methylparaben) was only used in gel diet (Moadeli et al., 2017). Moreover, the yeast concentration used in the gel diet was almost double that of the carrot diet (see Supplementary Tables S1, S2). Yeast and yeast like fungi are key sources of amino acids for developing larvae (Martin, 1987; Nardon and Grenier, 1989; Vega and Blackwell, 2005; Moadeli et al., 2018c). Previous studies found that Enterobacteriaceae support metabolic activities in *C. capitata* and *B. oleae* larvae and support nitrogen fixation and pectinolysis (Behar et al., 2008b; Ben-Yosef et al., 2015). It might be that larvae reared on the gel diet consumed more yeast and that the high abundance of Enterobacteriaceae was needed for protein hydrolysis (Pavlidi et al., 2017). Additionally, the gel diet had added cane sugar, while in the carrot diet, sugar was only as naturally occurring sucrose and glucose. This difference in sugar content of larval diets could underlie the presence of *Swaminathania/Asaia*. Acetobacteraceae has been reported to support break down and digestion of complex glucose structure and lipid content of the larval diet in *Drosophila* (Huang and Douglas, 2015). Before the domestication process began, the wild type larvae fed on host fruits. *Swaminathania/Asaia* would have likely been transferred with the larvae used to establish colonies and then become abundant in the larval microbiome of the colony reared on the carrot diet. Deutscher et al. (2018) and Majumder et al. (2019, 2020) made similar observations. We suggest that bacteria from the Acetobacteraceae may be needed for the digestion of complex carbohydrate and are not needed with gel-based artificial diet, which contains only cane sugar, hence their absence. Bacterial taxonomic composition in the carrot diet reared colony was similar to that of wild flies. In contrast, bacterial taxonomic composition was highly altered across all developmental stages of the Qfly reared on the gel based larval diet. It could also be that the presence of nipagin in the gel larval diet, in addition to sodium benzoate and citric acid, which is present in both larval diets, significantly alters the bacterial community.

The bacterial genus *Providencia* was abundant in all developmental stages of the Qfly reared on the gel diet but was absent in the carrot diet reared Qfly. *Providencia* is a gram-negative opportunistic, non-spore forming genus that is often pathogenic (O’Hara et al., 2000), and has also been observed and isolated from many other fruit fly species including *A. ludens* (Kuzina et al., 2001; Ventura et al., 2018), *A. obliqua, A. serpentina*, *A. striata* (Ventura et al., 2018), *B. oleae* (Kounatidis et al., 2009; Koskinioti et al., 2020), and *Z. tau* (Noman et al., 2020). Also, *Providencia* has been identified in domesticated *C. capitata* (Guerfali et al., 2018) and reported to cause infection. However, to date, there is no evidence of any pathogenic effect of *Providencia* in Qfly. Also, *Morganella* was highly abundant in the larval stages (∼99%) and less than 2% in other life stages of the Qfly fed on the gel diet, but was absent in the carrot diet fed Qfly (Figure 3). *Morganella* was first identified by Fulton (1943) and included in the family Enterobacteriaceae by Brenner et al. (1978). Salas et al. (2017) observed *M. morganii* as a lethal pathogenic bacterium in domesticated *A. ludens* larvae. Additionally, this pathogenic bacterial species has been detected in wild Qfly adults (Woruba et al., 2019) and in the larval stage of *Z. tau* (Noman et al., 2020). We found the same bacterial genera present in the larval microbiome from the Qflies reared on the gel diet. Although the gel diet contained more antimicrobial agents, *Morganella, Enterobacter, Citrobacter, Providencia* and *Burkholderia* were all detected at higher abundances in the Qfly fed the gel diet when compared to the carrot diet (Figure 3). *Morganella, Citrobacter*, and *Providencia* have been identified previously in wild Qfly adult (Woruba et al., 2019). We propose that, through continuous rearing on the same larval diet, these bacteria may develop a symbiotic relationship with the host and even contribute positively to the quality of Qfly across all developmental stages during laboratory rearing. We hypothesize that these bacterial genera might be controlled in part by the host genetics along with nutritional components present in the gel based larval diet (Noman et al., 2020). Other bacteria, found across all developmental stages of the Qfly fed on the gel diet, might follow the same trend. Additionally, given that these bacteria are found in the wild Qfly (Woruba et al., 2019), we hypothesize that these sometimes pathogenic bacteria genera may often be abundant as benign gut residents.

The relationship between the insect and its symbionts may be beneficial or harmful to the host health and fitness, and this depends on the overall microbiome composition (Kaufman et al., 2000; Marchini et al., 2002; Feldhaar, 2011; Hammer et al., 2017). Symbiotic and endosymbiotic bacteria can be important sources of nutrients to their host insects (Behar et al., 2005; Behar et al., 2008a). Further, the nutritional composition of the larval diet including yeasts and sugar, fatty acids and minerals has a strong influence on fruit fly development (Krainacker et al., 1987; Vargas et al., 1994; Aluja et al., 2001; Plácido-Silva et al., 2006; Moadeli et al., 2018a,b,c, 2020). In fruit flies, larval, pupal and adult body weight are commonly used measures of quality (Sharp et al., 1983; Churchill-Stanland et al., 1986; Fanson et al., 2014). We found that pupal weight was higher in Qfly from the gel diet. Pupal weight has been considered a key quality parameter in mass rearing of the Qfly (Dominiak et al., 2010). Greater pupal weight is generally expected to correspond to larger, healthier, adults (Mohamed et al., 2016). Our study is consistent with Mainali et al. (2019) who reported that the gel diet produced flies with better flight ability performance than the carrot diet. Moadeli et al. (2018c) reported that brewer’s yeast present in the gel diet is better for larval development than torula yeast that is used in carrot diet.

In tephritid flies, numerous studies have demonstrated that gut bacteria are associated with digestion, detoxification, immune response, metabolism, sexual behavior, reproduction, and survival (Behar et al., 2005; Hosokawa et al., 2007; Ben Ami et al., 2010; Augustinos et al., 2015; Ben-Yosef et al., 2015; Akami et al., 2019). Various strains of Enterobacteriaceae have been added to artificial larval diets to improve pupal weight and mating performance, and to decrease developmental time, in *B. oleae* and *C. capitata* (Sacchetti et al., 2008; Ben Ami et al., 2010; Gavriel et al., 2011; Hamden et al., 2013; Augustinos et al., 2015). Use of *Enterobacter* sp. AA26 and *Bacillus* sp. 139 in artificial larval diet increased pupal weight, increased adult survival under stress and improved adult production of *B. oleae* (Koskinioti et al., 2020). Furthermore, in *B. oleae* supplemented with *Providencia* sp. AA31 in artificial larval diet increased pupal and adult production, enhanced male survival under stress conditions and delayed immature development (Koskinioti et al., 2020). We found the same bacterial family of Enterobacteriaceae in the Qfly microbiome in both artificial diet groups at all developmental stages, but the Q-fly reared on the two different larval diets contained different bacterial genera. It might be that the presence of the different bacterial genera of the same family in Qfly fed the carrot or gel diet reflected the ability of these taxa to accommodate the diet-dependent changes in the gut environment during domestication from the wild. This accommodation may result in functional redundancy of some microbiome components, whereby some bacteria are replaced by others (pathogenic/non-pathogenic) with similar functions (e.g., improve metabolic activity or physiological development) (Moya and Ferrer, 2016).

The present study explored differences in the microbiome of Qfly reared, from wild origins, on two common artificial larval diets. Overall, we found that larval diet strongly effects the microbial community structure of the Qfly across developmental stages as well as key quality control parameters. This knowledge can guide development of larval diet formulation and manipulation of bacteria to improve quality of mass-reared Qfly for SIT.

## Data Availability Statement

The datasets presented in this study can be found in online repositories. The names of the repository/repositories and accession number(s) can be found in the article/ Supplementary Material.

## Author Contributions

RM designed the experiments, collected the data, and prepared the main draft of the manuscript. RM, BS, SA, and BM analyzed the data. TC and PT supervised the project. All authors provided inputs into the writing of the manuscript and approved the submitted version.

## Conflict of Interest

The authors declare that the research was conducted in the absence of any commercial or financial relationships that could be construed as a potential conflict of interest.

## Footnotes

1 https://doi.org/10.4225/08/59f98560eba25

## Supplementary Material

FIGURE S1 Artificial larval diet **(A)** Gel based diet **(B)** Carrot based diet.

TABLE S1 Gel based larval diet recipe.

TABLE S2 Carrot based larval diet recipe.

FILE S1 OTU table and Mapping file.
