# Supplementary figures and images for: Artificial Larval Diet Mediates the Microbiome of Queensland Fruit Fly
Source: Front Microbiol. 2020 Sep 16;11:576156. doi: 10.3389/fmicb.2020.576156 (PMC7526507; doi:10.3389/fmicb.2020.576156)

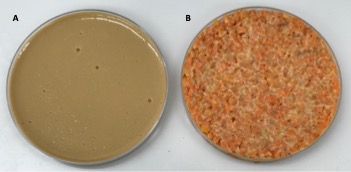

Supplement: FIGURE S1 — Artificial larval diet (A) Gel based diet (B) Carrot based diet. [file Image_1.JPEG]
