# Supplementary material for: Artificial Larval Diet Mediates the Microbiome of Queensland Fruit Fly
Source: Front Microbiol. 2020 Sep 16;11:576156. doi: 10.3389/fmicb.2020.576156 (PMC7526507; doi:10.3389/fmicb.2020.576156)
Supplement: TABLE S1 — Gel based larval diet recipe. [file Table_1.docx]

**SUPPLEMENTARY TABLE S1 |** Gel based larval diet recipe

| **Ingredients** | **1kg diet preparation** | **Company name and catalogue number** |
| --- | --- | --- |
| Brewer’s Yeast (g) | 204 | Lallemand Australia Pty Ltd, Edwardstown, SA, Australia |
| Sugar (g) | 121.8 | MP Biomedicals LLC, France, (Cat. n^o^02902978) |
| Agar (g) | 10 | Sigma Aldrich® St. Louis, MO, USA |
| Citric Acid (g) | 23.1 | Sigma Aldrich®, St. Louis, MO, USA |
| Nipagin (g) | 2 | Southern Biological, Knoxfield, VIC, Australia, (Cat n^o^ MC11.2) |
| Sodium benzoate (g) | 2 | Sigma Aldrich® St. Louis, MO, USA |
| Wheat Germ Oil (ml) | 2 | Melrose laboratories Pty Ltd, Australia |
| Water (ml) | 1000 | Milli-Q-water |
