# Supplementary material for: Artificial Larval Diet Mediates the Microbiome of Queensland Fruit Fly
Source: Front Microbiol. 2020 Sep 16;11:576156. doi: 10.3389/fmicb.2020.576156 (PMC7526507; doi:10.3389/fmicb.2020.576156)
Supplement: TABLE S2 — Carrot based larval diet recipe. [file Table_2.docx]

**SUPPLEMENTARY TABLE S2 |** Carrot based larval diet recipe

| **Ingredients** | **1kg diet preparation** | **Company name and catalogue number** |
| --- | --- | --- |
| Carrot (dehydrated diced) (g) | 280 | H.J. Langdon, Derrimut, VIC, Australia |
| Torula yeast (g) | 89.55 | H.J. Langdon, Derrimut, VIC, Australia, (Product code 45014) |
| Citric acid (g) | 13.43 | Sigma Aldrich®, St. Louis, MO, USA |
| Sodium Benzoate (g) | 3.72 | Sigma Aldrich®, St. Louis, MO, USA |
| Water (ml) | 1000 | Milli-Q-water |
